# Harnessing Selectivity and Sensitivity in Electronic Biosensing: A Novel Lab-on-Chip Multigate Organic Transistor

**DOI:** 10.1021/acs.analchem.0c01655

**Published:** 2020-06-02

**Authors:** Vitaliy Parkula, Marcello Berto, Chiara Diacci, Bianca Patrahau, Michele Di Lauro, Alessandro Kovtun, Andrea Liscio, Matteo Sensi, Paolo Samorì, Pierpaolo Greco, Carlo A. Bortolotti, Fabio Biscarini

**Affiliations:** † Dipartimento di Scienze della Vita, Università degli Studi di Modena e Reggio Emilia, Via Campi 103, 41125 Modena, Italy; ‡ Scriba Nanotecnologie S.r.l., Via di Corticella 1838, 40128 Bologna, Italy; § Laboratory of Organic Electronics, Department of Science and Technology, Linköping University, 601 74 Norrköping, Sweden; ∥ University of Strasbourg, CNRS, ISIS UMR 70068, Alleé Gaspard Monge, 67000 Strasbourg, France; ⊥ Center for Translational Neurophysiology of Speech and Communication, Istituto Italiano di Tecnologia, Via Fossato di Mortara 17-19, 44121 Ferrara, Italy; # Istituto per la Sintesi Organica e la Fotoreattività, CNR, Via Piero Gobetti, 101, 40129 Bologna, Italy; ∇ Istituto per la Microelettronica e Microsistemi, CNR, Via del Fosso del Cavaliere, 100, 00133 Roma, Italy

## Abstract

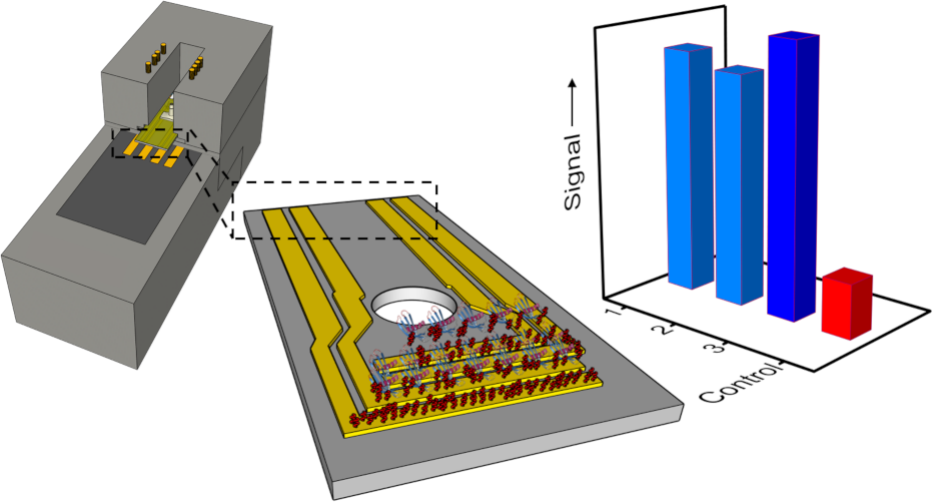

Electrolyte gated organic transistors can operate as powerful ultrasensitive biosensors, and efforts are currently devoted to devising strategies for reducing the contribution of hardly avoidable, nonspecific interactions to their response, to ultimately harness selectivity in the detection process. We report a novel lab-on-a-chip device integrating a multigate electrolyte gated organic field-effect transistor (EGOFET) with a 6.5 μL microfluidics set up capable to provide an assessment of both the response reproducibility, by enabling measurement in triplicate, and of the device selectivity through the presence of an internal reference electrode. As proof-of-concept, we demonstrate the efficient operation of our pentacene based EGOFET sensing platform through the quantification of tumor necrosis factor alpha with a detection limit as low as 3 pM. Sensing of inflammatory cytokines, which also include TNFα, is of the outmost importance for monitoring a large number of diseases. The multiplexable organic electronic lab-on-chip provides a statistically solid, reliable, and selective response on microliters sample volumes on the minutes time scale, thus matching the relevant key-performance indicators required in point-of-care diagnostics.

Different sensing strategies have been devised and employed for the quantification of pathological biomarkers. Most of these methods require the use of specialized laboratory environments, and only a few of them have become standard in clinical diagnostics. Platforms based on optical labels have demonstrated ultrasensitivity and versatility for identifying biomarkers through specific molecular recognition events, but they require expensive lab equipment and lack portability thus limiting applications in-field deployed and at the point-of-care.^1−4^ Optical sensing arrays and kits are commercially available to probe large numbers of samples, including control experiments. The latter are, de facto, necessary in order to assess the occurrence of nonspecific interactions and minimize the occurrence of false positives.

With the emergence of personalized medicine, there is a quest for accurate, selective, and reliable biosensors for point-of-care (PoC) applications, in environments where low cost, rapid response, and lack of specialized operators become stringent.^5,6^ Label-free organic electronic biosensors,^7,8^ which transduce recognition events or enzymatic activity into an amplified electrical signal, are emerging as novel tools for PoC diagnostics. With the recent progresses, these devices were shown to display a sensitivity comparable to those of established techniques such as enzyme-linked immunosorbent assay (ELISA) or surface plasmon resonance (SPR), with limits of detection (LOD) for biomarkers down to the aM range.^9−11^ Organic devices seem to be able to detect biomarkers at all length scales, from small molecules (like neurotransmitters) to viruses, to bacteria up to cells and tissues in vivo.^12−18^ Single-molecule binding of a biomarker (IgG) was reported, thus pushing the state-of-the-art LOD down to zepto-molar (10^–21^ M) scale.^19^ These results show the possibility to detect biological entities even at ultralow concentrations, thus opening possible applications to virtually any pathology, for which biomarkers have been identified.

Among the organic bioelectronics sensing platform, the electrolyte-gated organic field-effect transistor (EGOFET) is particularly powerful because it combines the technological advantages of organic electronics, such as cost-effectiveness and fabrication on flexible substrates, to the low voltage operation and ultrahigh sensitivity ensured by the electrolyte gating.^20,21^

Most of the EGOFET architectures reported in the literature operate with a single gate (G) electrode functionalized with a biorecognition moiety for analyte detection. Transfer characteristic curves (obtained by applying a voltage sweep *V*_GS_ at the gate-source terminals with fixed source-drain potential *V*_DS_ and measuring the current *I*_DS_ flowing in the source-drain channel) are used to assess the performance of the device. In order to quantify the analyte of interest in a given sample, it is always necessary to perform a blank test by running measurements also with the same gate driving the same buffer solution with no analyte and differentiate the parameters extracted from current responses in the presence and in the absence of the analyte.

The device instability due to bias stress^22−24^ upon repeated operations represents a potential drawback of EGOFET devices, which may also be resulting from the slow dynamics of ions inside the semiconductive channel.^25−27^ Another key issue in EGOFET is the specific recognition of the analytes of the interest. High precision in the detection of the binding events occurring exclusively at the gate-electrolyte interface is a must to boost selectivity in recognition processes. The presence of an analyte dispersed in liquid electrolyte, in which the gate electrode and semiconductors are immersed, may lead the occurrence of nonspecific binding at the electrolyte-semiconductor interface, thereby jeopardizing the device performance. Elegant and effective solutions based on ad hoc designed microfluidics and/or on the use of floating gate electrodes have been proposed as a route to minimize or suppress nonspecific binding events.^28−30^ Further improvement in the minimization of these potential artifacts in the signal by completely separating them from the signal response due to the biorecognition event is mandatory.

A further limitation in the use of current EGOFET architectures as biosensors stems from the use of a single gate device, and it is related to the precision of the response: a statistically solid result would require measuring the same sample at least in triplicate; this implies the use of single gate devices multiple times, by employing each time the required sample volume. The latter can represent a major drawback since sizable quantities of samples might be hardly available when measuring bodily fluids collected with invasive methods or stored in biobanks.

Here we propose a possible solution relying on a lab-on-a-chip layout, in which the same organic semiconductive channel is interfaced to multiple top gate electrodes. Such a device layout not only ensures high stability and control throughout the entire biosensing measurements, but it also guarantees a more robust statistical data set with respect to analyte detection executed using a single gate device. In a proof-of-concept experiment, we demonstrate the efficiency of our lab-on-chip device in quantifying the proinflammatory cytokine tumor necrosis alpha (TNFα). Detection of cytokines levels is of fundamental importance for real-time monitoring of the anti-inflammatory response every time the homeostasis of an organism is altered. In fact, human organisms restore the physiological conditions, after tissue injuries, bacterial or viral infections, or tumors, through the acute phase response, characterized by a strong disequilibrium in the production of cytokines (pro- and anti-inflammatory ones).^31^

Our multigate EGOFET is integrated with a single reservoir microfluidic system enclosed within a 3D-printed sample case holder (Figure 1d). We propose a new differential signal which highlights the contribution of specific recognition to the response: three gate electrodes enable simultaneous detection of TNFα with a LOD of 3 pM on a single sample; the fourth electrode serves as an internal reference, to assess whether the detected response has to be ascribed to the sensing event itself instead of other adventitious phenomena (nonspecific binding either at the gate or at the channel, else the drift due to bias stress) that could generate false positive or negative responses.

**Figure 1:**
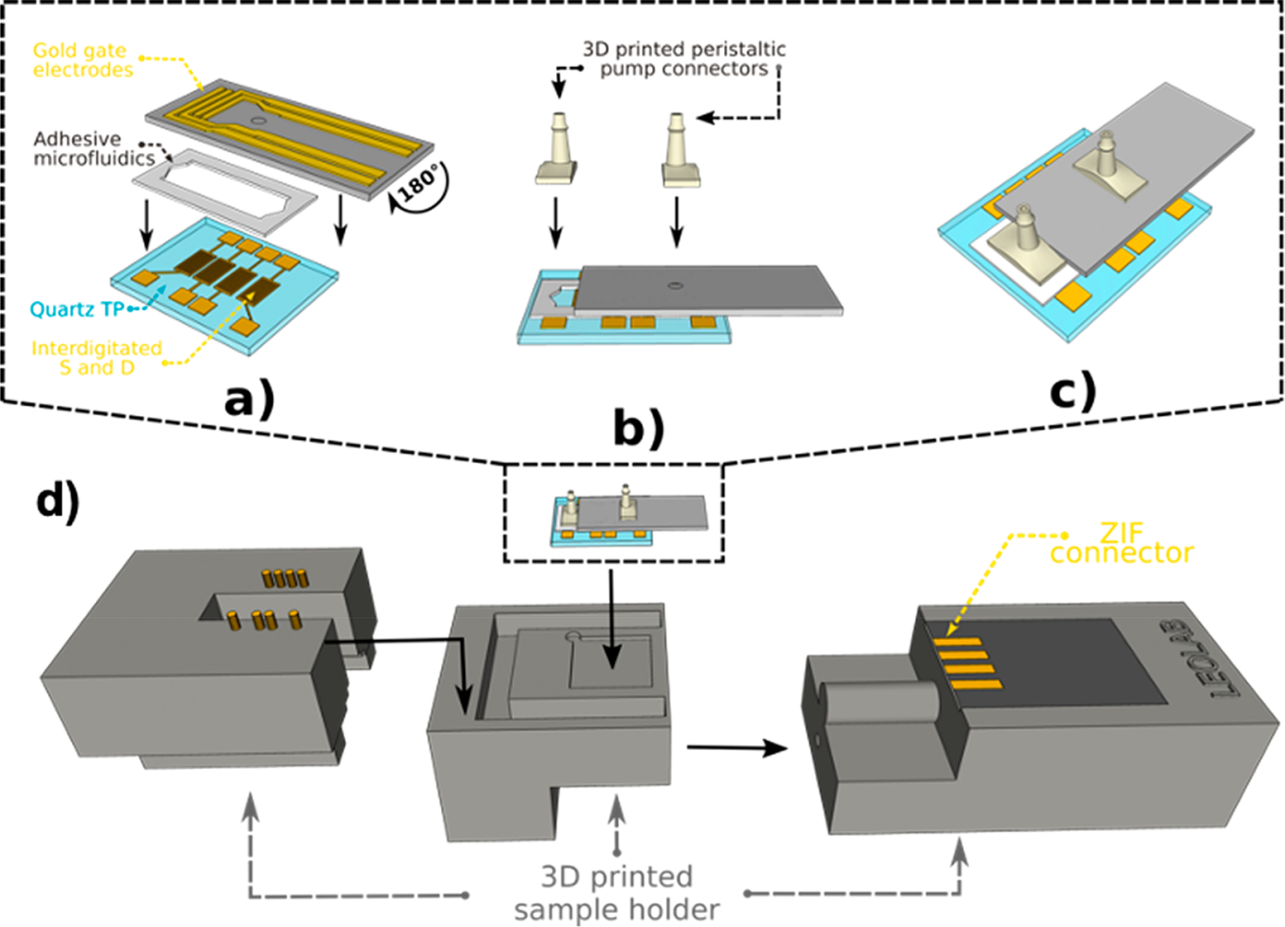
Schematic drawing of multigate sensor components (a) encompassing a quartz test pattern (TP) featuring Au source and drain interdigitated electrodes, an adhesive microfluidic chamber, four top gold gate electrodes, and (b) connectors for peristaltic pump tubes; (c) schematic picture upon assembly of the lab-on-chip. (d) Communication to the multiplexer and to the SMU is ensured by the ZIF connector.

## Experimental Section

### Gold Source and Drain electrodes

Interdigitated gold (Au) source (S) and drain (D) electrodes patterned on a 1 cm^2^ quartz substrate by photolithography and lift-off were purchased from “Fondazione Bruno Kessler” (FBK, Trento, Italy). The thickness of Au electrodes amounts to 50 nm with a few nm of Cr adhesive layer. Channel length and width amount to *L* = 15 μm and *W* = 30 mm, respectively, leading to a *W*/*L* = 2000. Each quartz test pattern (TP) comprises four sets of interdigitated S and D electrodes (Figure 1a).

The TP cleaning procedure consists of (i) device rinsing with 10 mL of acetone in order to remove the photoresist layer; (ii) gentle drying under nitrogen flow; (iii) washing in acetone at 80 °C for 15 min; and (iv) gentle drying under dry nitrogen flow.

### Semiconductor Thin Film Growth

The organic semiconductor of choice is pentacene grown in thin films by thermal sublimation in high vacuum (base pressure 10^–8^ mbar, deposition rate 2.5 Å/min) on the interdigitated electrodes with the substrate kept at RT during the film growth. The final pentacene film thickness is 15 nm (10 monolayers).

### Gold Gate Electrodes

**Figure 2:**
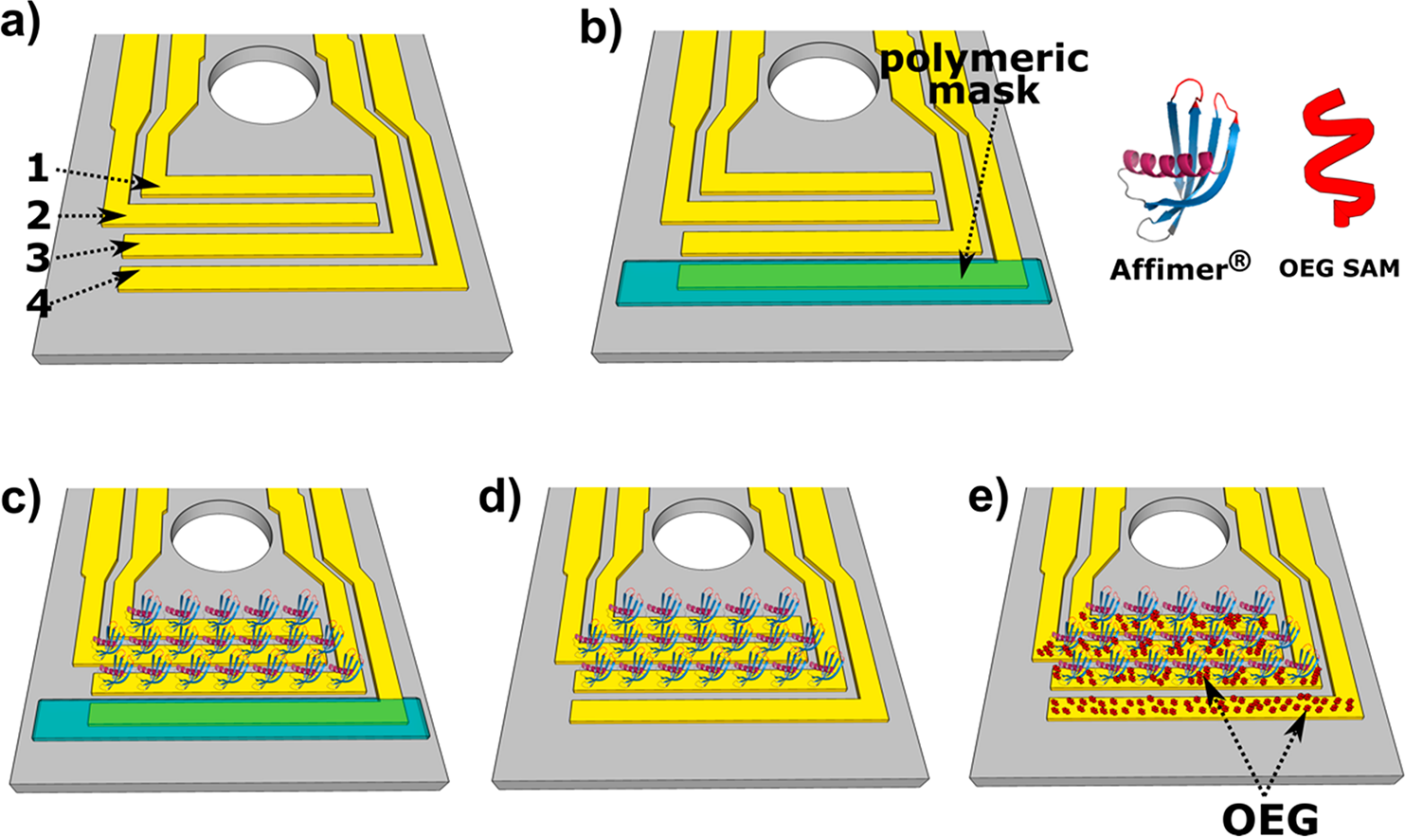
Gate electrodes functionalization strategy: (a) four bare Au electrodes; (b) one electrode is protected by means of a polymeric mask; (c) three electrodes are functionalized with anti-TNF**α** peptide aptamer (anti-TNFα Affimer); (d) the polymeric mask is removed by peeling; and (e) all four gate electrodes are functionalized by 11-mercaptoundecyl-triethylene glycol (OEG SAM). This step leads to the formation of a compact OEG self-assembled monolayer (SAM) on gate 4 and to the passivation of the gold spots eventually left uncoated on the other three electrodes.

A total of four gate electrodes were fabricated on a single 2.5 mm thick printed circuit board (PCB; FR4 glass epoxy) substrates with an electroless nickel immersion gold (ENIG) surface plating technique by Esseti S.r.l. (Bologna, Italy). The electrodes are 400 μm wide with 200 μm spacing between them (Figures 1a and 2). The design was chosen in order to provide a plug&play connection of all four gate electrodes by means of zero insertion force (ZIF) connector (Figure 1d). Gate electrodes were thoroughly sonicated for 15 min for each organic solvent as follows: (i) acetone, (ii) ethanol, (iii) isopropanol, and (iv) dried under nitrogen flow. They were rinsed with distilled water before final assembly.

### Gate Functionalization

The peptide aptamers used are Anti-TNFα Affimer (Avacta, U.K.). Affimers were treated with tris(2-carboxyethyl)phosphine hydrochloride (TCEP) immobilized on a dextran matrix to minimize any interprotein disulfide bonds (cysteine-cysteine bonds) and maximize the availability of free cysteine residues for immobilization on Au surfaces.^32,33^ A polydimethylsiloxane (PDMS) chamber (1.6 × 3.5 mm^2^) was placed on top of the electrodes in order to serve as a solution container during the functionalization steps: (i) incubation in anti-TNFα Affimer solution (0.25 mg mL^–1^) for 12 h at 5 °C; (ii) rinse with abundant buffer solution; (iii) incubation in 100 μg mL^–1^ triethylene glycol mono-11-mercaptoundecyl SAM (OEG SAM) solution for 20 min at room temperature; and (iv) final thorough wash with buffer solution.

### Reagents

Phosphate salts, acetone, ethanol, isopropanol, pentacene, and OEG SAM were purchased from Sigma-Aldrich. Reduction gel was purchased from Thermo Fisher Scientific. Recombinant human TNFα was produced by AdipoGen (Liestal, Switzerland) and purchased from Vinci-Biochem S.r.l. (Firenze, Italy). Cys-anti-TNFα Affimer was purchased from Avacta Life Sciences, Ltd. (Wetherby, U.K.).

### Microfluidic Chamber

In order to contain the electrolyte solution, a microfluidic chamber was sealed with two strips of double sided adhesive (each 225 μm thick), placed on top of each other with an intermediate (10 μm thick) polyethylene terephthalate layer (PET) layer, which served for further isolation to avoid any possibility of leakage of solution (Figure 1b,c). All of the parts of the microfluidic chamber were patterned with laser scan marker “Marko” (Laserpoint SRL, Milan, Italy) which has a pulsed (100 ns width, 20 kHz repetition rate, 50% duty cycle) Nd:YAG infrared (IR) laser centered at λ = 1064 nm.

Once assembled on the TP, the bottom part of the microfluidic chamber exposes all four pairs of interdigitated S and D electrodes covered with organic semiconductors (OSC); the upper part exposes the multiple-gate electrodes through a rectangular window (3.5 × 3.5 mm^2^) and provides their contact with electrolyte solution in order to successfully operate the EGOFET device. The manipulation of solution is made by means of a peristaltic pump, connected to the microfluidics through 3D printed connectors and necessary tubing.

### Support Structure and Connectors Fabrication

The modular support structure and the connectors for the peristaltic pump tubing were designed with Sketchup software and fabricated by use of B9Creator V1.2HD Digital Light Processing (DLP) 3D printer. “B9-Black” was the resin of choice with printing resolution on *x*, *y*, and *z* axis of 30 μm (Figure 1d).

### Electrical Characterization

Electrical measurements were acquired in buffer solution (PBS 10 mM, pH 7.4) injected into the microfluidic channel with a peristaltic pump (Watson Marlow 120D). Source and drain electrodes were connected to an Agilent B2902A Source-Measurement Unit (SMU).

The *I*–*V* transfer characteristic curves were acquired in the linear regime by sweeping the gate-source voltage (*V*_GS_) from −0.4 to −0.8 V while leaving the drain-source voltage (*V*_DS_) constant at −0.2 V potential. All measurements were carried out at room temperature by using a homemade custom multiplexer and custom control software.

### X-ray Photoelectron Spectroscopy (XPS)

High-resolution XPS spectra were obtained in ultra high vacuum condition (base pressure = 5 × 10^–9^ mbar) by using a Phoibos 100 hemispherical energy analyzer (Specs GmbH, Berlin, Germany) and Al Kα radiation (ℏω = 1486.6 eV; power = 125 W) in constant analyzer energy (CAE) mode. The probed area was about 1 mm diameter. Data analysis and fitting procedures were performed with CasaXPS software, after Shirley background subtraction. All of the spectra were calibrated at the binding energy (BE) of Au 4f_7/2_ eV (84.0 eV), setting the Au 4f doublet at fixed energy: BE(4f_5/2_) – BE(4f_7/2_) = 3.67 eV. In the case of S 2p, the energy difference of the doublet was set to BE(2p_1/2_) – BE(2p_3/2_) = 1.18 eV. Due to different chemical species, N 1s profile was fitted with two independent peaks.

## Results and Discussion

### Lab on Chip Layout

All of the measurements were performed in an in-house designed and assembled lab-on-chip encompassing a microfluidic adhesive chamber assembled on top of 15 nm-thick pentacene channel, thermally sublimed on Au interdigitated S and D electrodes. Four gold gate electrodes on a single glass-reinforced epoxy planar substrate were then assembled on top of the microfluidic chamber, to ensure simultaneous contact of all gates to the electrolyte solution (Figure 1b). The tubing connection to the microfluidic chamber was guaranteed by means of custom 3D printed connectors (Figure 1c), and the final device was assembled into a three-part custom 3D printed support, which featured a Zero Insertion Force (ZIF) connector for the gate electrodes and 8 spring contacts for top connection of the S and D electrodes (Figure 1d). The interfaces for connections to the source measurement unit (SMU) and to the gate electrode multiplexer were also developed in house. The final size of the lab-on-chip device, featuring an EGOFET as sensing core, were 7 × 3 × 0.25 cm^3^.

### Electrodes Selective Functionalization and Device Characterization

We decided to test the performances of our multigate EGOFET biosensor by detecting cytokine TNFα. Our lab has already demonstrated TNFα sensing down to pM regime with single gated EGOFET devices, using both anti-TNF**α** antibodies^34^ or peptide aptamers^35^ as recognition units. Therefore, TNFα sensors serve as a benchmark for our newly developed platform, allowing us to focus on the improvements brought into play by the multigate architecture rather than on the specific recognition problem.

We functionalized the four gates as follows (see Figure 2): first, three gates (1–3) were functionalized with a peptide aptamer (Affimer) selective toward TNFα, through covalent immobilization achieved by means of a single surface-exposed cysteine residue (step 1). During this functionalization step, the fourth gate electrode was protected by means of a polymeric mask.^36^ The polymeric mask was then removed by peeling (step 2), followed by incubation (step 3) of all four gate electrodes in a 100 μM aqueous solution of 11-mercaptoundecyl-triethylene glycol (OEG). This third step led to the formation of a compact OEG self-assembled monolayer (SAM) on gate 4, and to the passivation of the gold spots eventually left uncoated on electrodes 1–3, leading in these cases to bicomponent electrodes functionalization. The OEG was chosen because of its antifouling properties, in order to minimize nonspecific adsorption at the gate. The lab-on-chip device was then assembled with functionalized gate electrodes for electrical characterization, with each of the four gates individually addressed for transfer curve recording by means of a home-built multiplexer.

**Figure 3:**
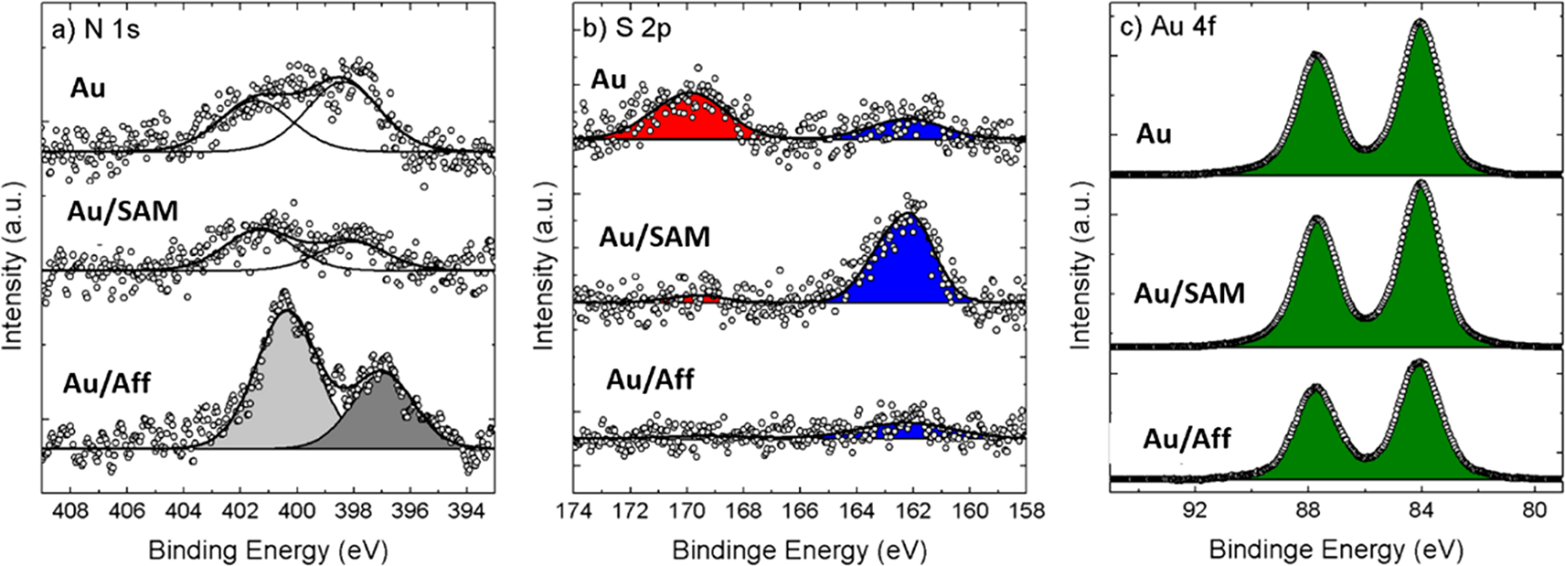
X-ray photoelectron spectroscopy analysis of (a) N 1s spectra, (b) S 2p spectra, red peak S–O group, blue peak S–Au, and (c) Au 4f spectra for bare Au electrode (Au), OEG SAM functionalized electrode (Au/SAM) and Affimer and OEG SAM functionalized electrode (Au/Aff).

The functionalization of the four gates was assessed first by X-ray photoelectron spectroscopy (XPS; Figure 3). The presence of Affimer and OEG SAM bound to the Au gate surface was confirmed by XPS analysis by monitoring the presence of gold (Au 4f), nitrogen (N 1s), and Sulfur (S 2p) contributions in the measured spectra (Figure 3). In particular, XPS has been performed on (i) pristine Au, (ii) Au functionalized with OEG SAM (Au/SAM), and (iii) Au functionalized with anti-TNFα Affimer (Au/Aff).

Pristine gold shows a residual amount of N and S contaminants, which can be resulting from the exposure to air. In particular, two S 2p peaks have been observed: S–O in the region 170–167 eV and thiol at c.a. 162 eV (red and blue areas in Figure 3b, respectively).

Despite of the low level of signal-to-noise ratio, the formation of a thiol-based SAM is confirmed by the S 2p peak located at ca. 162.0 eV in the Au/SAM spectra, in agreement with values reported in literature.^37^ Differently, in the case of Affimer a similar analysis has been not possible because of the thickness of the Affimer resulted similar to the penetration depth of the photoelectron (≲10 nm). Moreover, the presence of only one S atom in each Affimer molecule, surrounded by thousands of C, O, and N atoms poses an intrinsic problem related to the sensitivity of the XPS instrument. For this reason, only a small contribution has been measured (blue peak in Au/Aff spectra in Figure 3b) and the direct analysis of the peptide has then been performed using the N peak.^38^ To this end, two N 1s peaks has been measured on Au/Aff indicating the presence of two, at least, different nitrogen chemical states (i.e., R=N–H and R_2_–N–H), due to the chemical forms of N in the amino acidic chain of Affimer; in particular, the peak at 400.3 eV was associated with R–N–H_2_ groups,^39,40^ while the second peak at 397.0 eV was associated with other organic nitrogen. The signal from Au 4f decreases in samples with SAM and Affimer (Figure 3c) with respect to bare Au, confirming a substantial coverage in both cases.

### Multigate EGOFET: Analysis of the Transfer Curves

**Figure 4:**
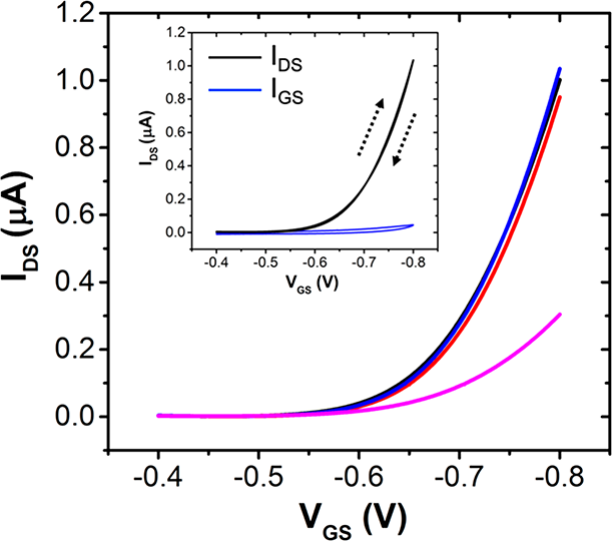
Comparison of the transfer characteristics of the EGOFET-based sensor registered with the four gate electrodes. Gates 1, 2, and 3 are functionalized with anti-TNFα Affimer and OEG SAM (black, blue, and red curves), while gate 4 with OEG SAM only (pink). Inset: forward and backward transfer curve registered for one gate electrode functionalized with anti-TNFα Affimer and OEG SAM in 10 mM PBS.

The transfer characteristics recorded for the four gate electrodes are compared in Figure 4. The three gate electrodes functionalized with anti-TNF**α** Affimer (gates 1–3) yield superimposable transfer curves, which, on the same time, are markedly different from the transfer curve of gate 4, the latter being passivated only with OEG SAM. In particular, for gates 1–3 the maximum *I*_DS_ at *V*_GS_ = −0.8 V is about 1 μA, while for gate 4 is as low as 0.3 μA, viz. three times lower. Transconductance *g*_m_ also shows marked differences: *g*_m_ = 1.91 ± 0.07 μS vs 0.59 ± 0.02 μS for Affimer-functionalized gates 1–3 and 4, respectively. Interestingly, the threshold voltage *V*_th_ is the same for gates 1–3 (*V*_th_ = −682 ± 4 mV) and gate 4 (*V*_th_ = −680 ± 1 mV). These combined data, combined a marked difference in the transconductance together with the invariance of the threshold voltage, suggest that the nature of the different signals for electrodes 1–3 with respect to electrode 4 has a capacitive nature since the electrochemical potential of the organic semiconductor (which is associated with charge transfer) is unaltered. The different capacitance can be ascribed to the surface chemistry at the two electrode sets: in the case of gate 4, the capacitance is mostly resulting from the packed OEG SAM; in the case of gate 1–3 the surface inhomogeneity due to the presence of Affimers alternated to the OEG-SAM leads to a reorganization of the water dielectric layer at the interface and possibly allows the penetration of water molecules and ions closer to the surface. The capacitance of this leaky capacitor, being no longer dominated by the OEG-SAM, also contains contributions from other in-parallel capacitances.

### Multigate EGOFET: Statistics and Internal Reference within a Single Device

**Figure 5:**
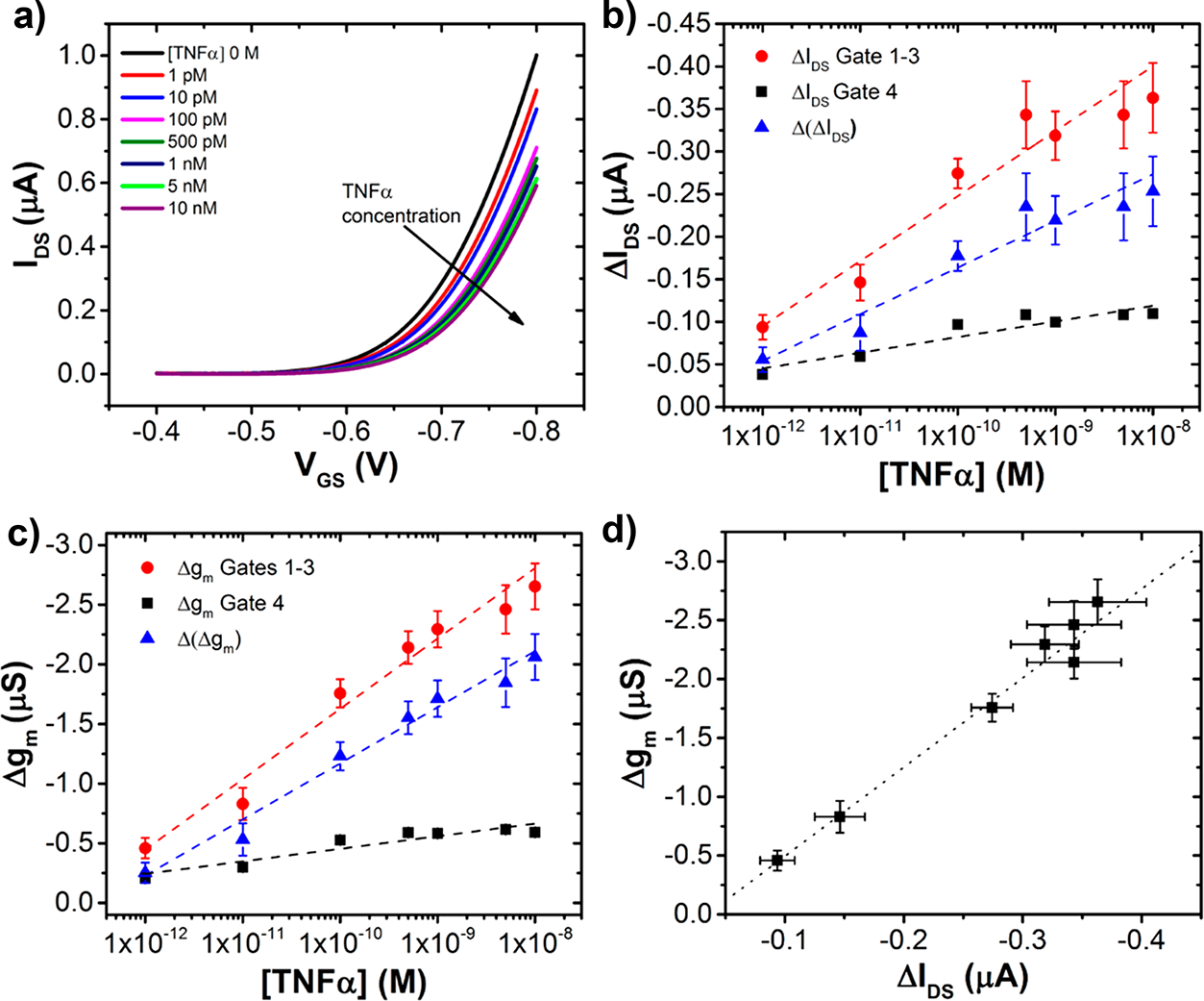
(a) Transfer characteristics of EGOFET biosensors upon exposure to different concentrations of TNFα in PBS buffer. The corresponding TNFα concentrations are reported in the legend. (b) Variation of output current as a function of TNFα concentration, acquired at *V*_GS_ = −0.8 V for sensing gates (red circles) and reference gate (black squares), Δ(Δ*I*_DS_) is the genuine contribution of the specific recognition to the sensor response (blue triangles). Data are fitted with eq 1. (c) Variation of transconductance *g*_m_ as a function of TNFα concentration for sensing gates (red circles) and reference gate (black squares); Δ(Δ*g*_m_) is the genuine contribution of the specific recognition to the sensor response (blue triangles). Data are fitted with eq 1. The error bars correspond to the rms of the signal averaged over three sensing electrodes. (d) Correlation plot between variation of output current and variation of transconductance for the sensing gates.

For building the dose curve, aqueous solutions containing increasing concentrations of TNFα, ranging from 1 pM up to 10 nM, were injected in the lab-on-chip microfluidics chamber and the transfer characteristics were acquired after each injection. At each concentration, the four gates were simultaneously exposed to the aqueous sample, although the transfer characteristics were recorded individually while the other gate electrodes were kept floating. The typical response of the EGOFET to increasing TNFα concentration, recorded for gate 1, is shown in Figure 5a. Similar trends were observed for gates 2 and 3 (see below). As previously observed for EGOFET-based TNFα biosensors,^34,35^ the current I_DS_ decreases for increasing concentration of TNFα.

We monitored changes in the drain current as a function of the TNF**α** concentration. In particular, we calculated the current change at different TNFα concentrations as Δ*I*_DS_ = *I*_DS(n)_ – *I*_DS(0)_. *I*_DS(n)_ is the drain current value at the *n*th TNFα concentration at *V*_GS_ = −0.8 V, and *I*_DS(0)_ is the drain current value when [TNFα] = 0 M, i.e., in pure buffer, also taken at *V*_GS_ = −0.8 V.

Figure 5b portrays the plot for Δ*I*_DS_ values averaged out over gates 1–3, with the corresponding standard deviation defining the associated error bar. It reveals that the curves acquired with the three Affimer-functionalized gates follow the same trend in response to the increasing TNFα concentration, with standard deviations never exceeding 15% of the mean value. This finding indicates that the reproducibility of the functionalization process allows the three gates to be used to simultaneously perform [TNF**α**] quantification in triplicate, thus assessing the precision of the assay.

For gate 4, functionalized with OEG SAM only, the ΔI_DS_ changes occurring vs [TNF**α**] are much smaller, never exceeding 0.1 μA (which is approximately the ΔI_DS_ obtained by gates 1–3 at [TNFα] = 1 pM, i.e., the lowest concentration used). The value 1 pM indeed compares to the LOD of our device, that we calculated as the concentration corresponding to the mean blank signal value (absence of TNF**α**) + three times the associated standard deviation, equal to 3 pM.^41^ The well distinct behavior of the gate 4 validates its effective role as an internal reference electrode.

Previous works^13,17,34,35,42^ suggested that the response of EGOFET biosensors with passivated functionalized gate electrodes is mainly determined by changes in the effective capacitance. This also seems being the case in the present work: in Figure 5c, we display the Δ*g*_m_ = *g*_m(n)_ – *g*_m(0)_, with *n* and 0 identifying the values at a given [TNFα] value and for [TNFα] = 0. The lin/log plots of Δ*g*_m_ vs [TNFα] for gates 1–3 and 4 closely resemble those by Δ*I*_DS_ vs [TNFα]. This is confirmed by the correlation plot shown in Figure 5d. Transconductance *g*_m_ embodies the product of the charge mobility μ and the capacitance; if we assume that charge carrier mobility does not undergo sizable changes upon TNFα binding,^20^ the changes of *g*_m_ upon biorecognition events can be ascribed mostly to effects on the capacitance.

The changes of the drain current ΔI_DS_ measured from the transfer characteristics of gate 4 (Figure 5b) may arise from nonspecific adsorption, either at the gate or at the OSC. Since they are much smaller than the changes detected at the functionalized gates 1–3 at any concentration explored, we treat them as additive independent contributions that we can subtract from the current response obtained with gates 1–3. Hence, in order to minimize the contribution of the nonspecific events in the change of the drain current ΔI_DS_, we propose to calculate at each concentration the Δ(Δ*I*_DS_) = Δ*I*_DS,(gate 1–3)_ – Δ*I*_DS,(gate 4)_ curve. The resulting values are depicted in Figure 5b.

Similarly, we extracted the Δ(Δ*g*_m_) values whose trend as a function of [TNFα] is reported in 5c.

We believe that the Δ(Δ*I*_DS_) parameter, obtained by subtracting the drain current value from gate 4, represents the genuine contribution of the specific binding to the device response. The plot of the Δ(Δ*I*_DS_) vs [TNFα] in the semilog format features an approximate linear trend, we therefore decided to fit it using the following equation:
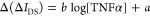
1

In this equation, the parameter *b* defines the sensitivity of the sensor and its value derived from the best-weighted fit is 55 ± 7 nA/decade. We can fit using the same equation the plot of Δ*I*_DS_ vs [TNFα]: the resulting *b* value is 76 ± 7 nA/decade. Similar fitting of Δ(Δ*g*_m_) and Δ*g*_m_ yields the *b* values −0.472 ± 0.007 μS/decade and −0.595 ± 0.007 μS/decade, respectively. The values of Δ*V*_th_ and Δ(Δ*V*_th_) are more scattered (data not shown) and we do not fit them.

The device sensitivity when using the Δ(Δ*I*_DS_) as sensing parameter is about 30% lower from the sensitivity extracted from the total signal. This decrease in sensitivity is less pronounced (about 20%) when using the *g*_m_ as a parameter. Nevertheless, we believe that the proposed data treatment process allows to discriminate the genuine contribution of the specific recognition to the sensor response.

## Conclusions

A novel lab-on-chip device integrating a four-gated EGOFET has been fabricated and exploited as biosensing unit for label free detection of inflammatory biomarker TNFα with a sensitivity down to the pM regime. Through selective functionalization of the gate electrodes with specific peptide aptamer and antifouling SAM, the electronic biosensor provides measurement in triplicate and simultaneous identification of nonspecific response thanks to the presence of an internal reference electrode. We show that a purely selective signal can be extracted out of the device, thus making it possible to quantify the genuine selectivity of the device toward the target analyte. In particular, the removal of the contribution of nonspecific binding events decreases the sensitivity by a few tens percent, still enabling a highly selective detection of the analyte and the control on possible artifacts. This is verified across 5 orders of magnitude of concentration of an important inflammation biomarker, i.e., TNFα. The choice of EGOFET as core transduction element ensures a label free response on the few minutes time scale. Through the integration of the multigate architecture with a 6.5 μL microfluidic channel, our device represents a major step forward in terms of robustness and cost-effectiveness of the assay, reduces the device fabrication effort and most importantly meets a central requirement of diagnostics (also, though not restricted, to field deployment use) as it enables to increase statistics on biomarker detection within a minimum sample volume. Parallel gates architecture may be exploited both for point-of-care and in-field diagnostics: with a small sized battery (operability potential <1 V), actuating also the fluidics compartment, integrated chip (IC) for signal analysis and an optional display, tests with picomolar sensitivity will be available for large distribution, like glucose sensors or lateral flow immunoassays. The fabrication process of the device allows integration on various substrates, even flexible polymeric substrates with the aim to reduce the overall size, increase production throughput with roll-to-roll technology, and reduce the costs of production by spotting the antigen functionalization. Moreover, as the multiple gates can in principle be functionalized individually with different biorecognition moieties through bioprinting, the presented lab-on-chip also paves the way for on-demand multiplex detection of a large portfolio of different analytes.
